# Pre-trained Artificial Intelligence Models in the Prediction and Classification of Atherosclerotic Cardiovascular Disease

**DOI:** 10.5152/eurasianjmed.2025.25937

**Published:** 2025-10-22

**Authors:** Furkan Şakiroğlu, Cemil Çolak, Mehmet Cengiz Çolak

**Affiliations:** 1 Biostatistics and Medical Informatics, Atatürk University Faculty of Medicine, Erzurum, Türkiye; 2 Biostatistics and Medical Informatics, İnönü University Faculty of Medicine, Malatya, Türkiye; 3 Cardiovascular Surgery, İnönü University Faculty of Medicine, Malatya, Türkiye

**Keywords:** Atherosclerotic cardiovascular, deep learning, machine learning, medical imaging, pre-trained artificial intelligence, risk prediction

## Abstract

Atherosclerotic cardiovascular disease (ASCVD) is one of the leading causes of global morbidity and mortality. The current study provides a systematic review of the use of artificial intelligence (AI) technologies applied to the prediction and management of ASCVD. Traditional risk assessment approaches have their restrictions, leading to a growing preference for AI and machine learning techniques in risk assessment.

First, this study tackles the complex pathophysiology of ASCVD and the problems associated with the current diagnosis, followed by an in-depth analysis of the wide variety of AI models that can be applied to electronic health records, medical imaging data, and other biomarkers. Special attention will be paid toward the potential of natural language processing models like bidirectional encoder representations from transformers in predicting risk from textual clinical data, and the overwhelming success of convolutional neural networks such as residual neural network and visual geometry group in plaque-based analysis through imaging modalities.

Although the research results show that these models have a lot to offer in the clinical world, the authors also describe some serious disadvantages: data bias, interpretability of the model, and computational needs. It highlights, in particular, the need for multicenter validation studies as well as developing explainable AI techniques.

Overall, AI-based approaches may pave the way for a new paradigm inASCVD management. Nevertheless, deploying these technologies in everyday clinicalpractice will require overcoming technical, ethical, and regulatory challenges. As such, interdisciplinary collaboration and thorough clinical validationstudies are essential for fulfilling the promise of these novel strategies to enhance patient outcomes.

**Main Points**

- Pre-trained models like bidirectional encoder representations from transformers, (text) and convolutional neural networks (residual neural network, visual geometry group) show potential in atherosclerotic cardiovascular disease (ASCVD) risk prediction, improving diagnostic accuracy and workflow efficiency.
- Med-BERT outperform models without pre-training, overcoming data limitations through transfer learning on electronic health records.
- The CNNs excel in plaque detection, improving the diagnosis of coronary artery disease and other ASCVD-related tasks.
- Issues include biases in training data, the “black box” nature, and the need for interpretability.
- Focus on reducing bias, improving interpretability, and integrating multi-modal data for better ASCVD prediction.

## Introduction

Atherosclerotic cardiovascular disease (ASCVD) encompasses a range of conditions, including coronary heart disease, cerebrovascular disease, peripheral artery disease, and aortic atherosclerosis, all resulting from plaque buildup in arterial walls.^1^ This pathological process significantly contributes to global morbidity and mortality.^2^ Early and accurate diagnosis of coronary atherosclerosis is crucial to preventing fatal outcomes and minimizing healthcare costs.^3^ Accurate disease prediction enables timely interventions to reduce adverse cardiovascular events.^4^ Although risk assessment models like QRISK3 and the ASCVD risk score are widely used, they often fail to fully capture the complex and nonlinear relationships among risk factors, limiting their applicability across diverse populations.^5^ Given the multifactorial nature of ASCVD, which involves intricate interactions between biological and lifestyle variables, there is a growing need for more sophisticated models that utilize nonlinear methodologies instead of traditional linear ones.^6^

Artificial intelligence (AI), particularly machine learning (ML), has emerged as a transformative tool in cardiovascular medicine.^7^ The AI-based systems now play an increasing role in detecting and predicting ASCVD risk.^8^

As cardiovascular diseases remain the leading cause of global health burden, especially in aging populations, their associated costs strain healthcare systems significantly.^9^ Despite advances in therapeutic interventions, uncertainty remains regarding risk factor variability across novel cardiovascular conditions. Machine learning—especially deep learning (DL)—is increasingly recognized for its potential to detect cardiovascular diseases more effectively. Artificial intelligence integration in early diagnosis could enhance population-level outcomes and reduce healthcare expenses. Ongoing research explores DL and reinforcement learning applications, with promising results such as detecting multiple cardiovascular conditions from a single imaging modality.^10^

## Deep Learning Approaches for Atherosclerosis Detection

### Histopathology-Based Deep Learning Models

DeepAD is a histopathology-based DL model that uses semantic segmentation networks to detect atherosclerotic lesions from intravascular optical coherence tomography (OCT) images.^11^ This underscores the necessity of expert-validated training data for optimal accuracy. While DeepAD can detect calcified lesions, its intersection over union (IOU) for this task is lower (0.34), often localizing calcifications but underestimating their size. Clinically, it shows high concordance with expert manual evaluations, with 88% agreement across 3284 frames and solid performance metrics: 86.8% sensitivity, 82.9% specificity, and 85.8% accuracy.^11^ It performs pixel-level classification and achieves a median IOU of 0.68  ±  0.18 on test patients.

A key strength of DeepAD lies in its training with histopathology-annotated data. Excluding these annotations reduces performance significantly—dropping IOU from 0.68 to 0.42, a 33% decrease.^11^

### Transfer Learning for Enhanced Detection

Deep learning architectures, particularly convolutional neural networks (CNNs), have been widely applied to ASCVD prediction, detecting complex patterns in high-dimensional data such as medical images and electronic health records (EHRs). For instance, a residual CNN combined with a discrete-time survival loss function outperformed traditional risk models and the pooled cohort equations (PCE) in predicting 10-year ASCVD risk.^12^ Transfer learning has also demonstrated high accuracy (>96%) in predicting cardiovascular events,^13^ particularly through the fine-tuning of pre-trained CNNs for plaque detection.^14^ This is especially valuable given the limited availability of large, annotated cardiovascular imaging datasets. Pre-trained models, adapted from large-scale image databases, have been effectively repurposed for cardiovascular tasks. In coronary computed tomography (CT) coronary CT angiography (CCTA) imaging, a DL model achieved 94% sensitivity and 90% accuracy in classifying calcified, non-calcified, and mixed plaques.^15^ Such models not only improve diagnostic precision butalso save time and expertise needed for manual image analysis.

These models enhance diagnostic accuracy while reducing the need for manual analysis. Similarly, in intravascular OCT, pre-trained DL tools enable rapid 3D visualization of atherosclerotic lesions, potentially improving clinical decision-making and treatment planning through intuitive disease representation.^11^

### Hybrid and Ensemble Methods

To enhance prediction accuracy, hybrid AI models—such as Artificial Neural Network combined with Genetic Algorithm (ANN-GA) and ensemble learning—have been proposed, achieving prediction rates as high as 96.56%.^16^^-^^18^ These models integrate multiple algorithms, leveraging their complementary strengths.

Pre-trained models have also been enhanced through multi-modal data integration, incorporating electrocardiograms (ECGs), medical imaging, and clinical variables.^19^ Notably, a self-supervised learning approach combining ECG and magnetic resonance imaging (MRI) data from one of the largest cardiovascular imaging datasets improved balanced accuracy by 7.6% compared to traditional supervised models.^20^ The integration of diverse data sources in multi-modal models significantly strengthens risk prediction and assessment.

### Genetic and Multi-Omics Strategies

Recent studies have integrated genetic and multi-omics data into AI models to improve ASCVD prediction. Machine learning frameworks that include qualitative and quantitative plaque features from CT angiography have outperformed traditional methods in identifying individuals at risk of rapid plaque progression.^21^ Similarly, models incorporating gut microbiota signatures have been developed to guide personalized treatment strategies, highlighting the potential of multi-omics in ASCVD management.^22^

Personalized risk prediction is increasingly supported by pre-trained AI models. For example, the PowerAI-CVD model, developed for the Chinese population, combines physiological, clinical, and laboratory data to predict major adverse cardiovascular events (MACE) with an area under the curve (AUC) of 0.869.^23^ Such models support clinicians in tailoring interventions to an individual’s specific risk profile.

### Artificial Intelligence Models forCardiovascular Risk Prediction

- a. Risk factor analysis

Deep learning has been applied to large-scale datasets to explore complex and unimaginable risk factors related to ASCVD; for example, variability of blood pressure, metabolism, and other surrogate markers. These models assist in identifying high-risk individuals and designing personalized treatment plans.^24^

b. Transfer learning for self-attention models

Beyond detecting existing atherosclerotic plaques, AI models are increasingly being trained to predict future cardiovascular events. For instance, Li et al^23^ demonstrated that a self-attention-based model using time-series features from electronic medical records could forecast MACE within a 3-year window.^9^ This highlights the value of transfer learning, particularly for institutions with limited data, by employing code mapping and feature selection strategies.

Transfer learning significantly improved model performance, increasing the AU the receiver operating characteristic curve (AUROC) from 0.564 to 0.821.^9^ Researchers also standardized definitions for diagnoses, medications, and lab tests to streamline heterogeneous codes, reduce feature complexity, and enhance interpretability and data quality across hospital systems.^9^

### Machine Learning Classification Model

Machine learning algorithms have shown notable success in classifying valvular heart disease, a leading cause of mortality, particularly in older adults. A 2-stage approach involving feature extraction—using a wrapping method refined by logistic regression—and classification via 5 algorithms (ANN, XGBoost, RF, Naive Bayes, and SVM) has been employed.^8^

To address small datasets and class imbalance in cardiovascular prediction tasks, advanced techniques such as stratified k-fold cross-validation and synthetic minority over-sampling technique have been applied, improving model accuracy and predictive performance.^8^ Artificial intelligence models are also used for ASCVD risk stratification, including the prediction of cardiac mortality and MACE. One study found that AI-based coronary artery calcium scans outperformed traditional biomarkers like NT-proBNP in predicting heart failure.^25^ Additionally, joint models incorporating longitudinal risk factor data have surpassed conventional models such as the PCEs in estimating 10-year ASCVD risk.^26^

### Multi-Modal Imaging Approaches

- a. Cardiovascular imaging

Artificial intelligence has been increasingly applied to cardiovascular imaging,^9^ particularly using CT to characterize atherosclerotic coronary artery disease (CAD). Deep learning models, such as CNNs, facilitate lesion detection, segmentation, and classification by extracting detailed information on coronary inflammation and plaque composition.^27^^,^^28^

Imaging techniques like CCTA and cardiac MRI provide valuable insights into plaque morphology. Pre-trained AI models applied to these datasets have been effective in plaque detection, disease progression prediction, and identification of high-risk patients. For example, Laidi et al (2022)^29^ reported that a DL model using CCTA achieved 95.2% accuracy, with an 11% increase in sensitivity through Haar wavelet decomposition. Transfer learning models with MRI data have achieved an overall high accuracy (97.94%-98.08%) in cardiovascular disease diagnosis.^30^

b. Retinal imaging for cardiovascular risk assessment

A novel approach to cardiovascular risk assessment is combining retinal imaging with AI analysis. On a different research path, AI is being integrated with non-invasive retinal scanning to better study heart function and predict changes in heart status based on the microvasculature.^9^^,^^31^ Through fundus retinal images, models have been trained to predict scores for carotid atherosclerosis and optimize the process of prediction.^10^

This non-invasive method uses the retina as a window to observe microvascular health, which could mirror systemic vascular states. This ability to detect precursors of atherosclerotic diseaseduring regular eye exams is a new frontier in preventive cardiology.

c. OCT-based cardiovascular assessment

Limitations of conventional coronary angiography have led to the increased use of OCT for detailed assessment of atherosclerotic lesions. Combined with DL algorithms like DeepAD, OCT can detect lesion characteristics such as calcification and improve segmentation and classification accuracy.^11^^,^^32^ In clinical cohorts receiving both coronary angiography and intravascular imaging, DL has facilitated rapid identification of diseased vessel segments.^11^

These models enhance plaque characterization by differentiating fibrolipidic and calcified lesions, offering greater precision in risk stratification and therapeutic planning.^11^ Pre-trained AI models using transfer learning have further advanced early diagnosis and risk prediction, with the potential to reduce global cardiovascular mortality.^33^

They are changing how risk stratification, disease diagnosis, treatment selection, prognostication, and prediction are approached, all aimed at improving patient outcomes. The AI/ML’s potential to enhance diagnostic accuracy, predictive ability, workflow efficiency, and resource use in cardiovascular disease management is increasingly recognized.^7^ The AI/ML-based predictive models can transform health systems by predicting disease pathways, identifying at-risk populations, and optimizing treatment under precision medicine principles. Additionally, AI algorithms using multi-modal imaging data like MRI, CT, and echocardiography are valuable in diagnosing cardiac diseases, providing risk factor information such as heart failure or CAD.^34^ By using advanced algorithms, AI can process vast amounts of data with unmatched accuracy, leading to earlier diagnoses, accurate risk assessments, and personalized treatments.^7^

A key innovation in AI is pre-trained models, particularly in DL.^35^ Transfer learning, where knowledge from large datasets is applied to new, related tasks, is crucial.^36^ Pre-trained models, such as CNNs trained on general-purpose datasets like ImageNet, can be adapted for medical image analysis through fine-tuning, reducing the need for costly annotated medical datasets.^37^ Large models like bidirectional encoder representations from transformers (BERT), pre-trained on text corpora, produce contextual embeddings that can enhance performance in smaller task-based datasets.^38^ Transfer learning helps improve models with limited data by adapting to dataset differences,^39^ mitigating concerns about data scarcity and annotation costs. Among pre-trained models, BERT have achieved success in natural language processing (NLP).^40^ The BERT’s embeddings can improve cardiovascular disease datasets, especially through fine-tuning on smaller datasets, enhancing diagnostic accuracy. Residual neural networks (ResNets) use residual connections to address the vanishing gradient problem, making them ideal for complex image analysis like atherosclerosis detection. Visual geometry group (VGG) networks, another CNN type, are often used in transfer learning with medical images for atherosclerosis detection.^41^ The BERT is suited for clinical text data, while ResNet and VGG excel in medical image detection and classification.

This review synthesizes research on the use of pre-trained AI models for predicting and classifying ASCVD. With AI’s growing role in cardiovascular care and the global burden of ASCVD, this review is crucial.^7^ By critically appraising existing methods, results, and limitations, it informs researchers, clinicians, and stakeholders on the field’s current state, highlights knowledge gaps, and stimulates future research and clinical advancements.

## Literature Search Methodology and Selection Criteria

This review will implement a comprehensive search strategy across 3 major academic databases, namely: Google Scholar, PubMed, and Scopusto ensure an in-depth and systematic extraction of relevant academic literature. A wide search will be carried out on multiple disciplines and sources, such as scholarly articles, theses,books and abstracts using Google Scholar, with an emphasis on finding emerging research and potentially missed grey literature. To access the largest collection of citations for biomedical literature, a postgraduate research meeting of the application will search through PubMed—an information source from theU.S. National Library of Medicine—giving access to a wide range of information relating to studies in medicine and public health. Scopus is ahuge database of peer-reviewed literature which includes scientific, technical, and medical journals, books, and conference proceedings and will give insight into the scientific/medical research output across multiple disciplines. The literature search across 3 databases will be based on the fundamental search string:“Pre-trained Artificial Intelligence Models in Atherosclerotic Cardiovascular Disease.” To further broaden the scope and capture a wider array of relevant studies, additional keywords and combinations will be employed, including “Pre-trained AI models ASCVD prediction,” “Pre-trained deep learning models atherosclerosis classification,” “BERT cardiovascular risk,” “ResNet atherosclerosis detection,” “VGG coronary artery disease AI,” “Transfer learning cardiovascular disease prediction,” and “Fine-tuning AI models cardiology,” along with combinations of terms such as “Atherosclerosis,” “Cardiovascular Disease,” “Coronary Artery Disease,” and “Stroke” with “Artificial Intelligence,” “Machine Learning,” “Deep Learning,” “Pre-trained Models,” “BERT,” “ResNet,” “VGG,” “Prediction,” and “Classification.” The search will be primarily limited to studies published in the English language within the last 5-10 years to ensure the inclusion of the most recent advancements in this rapidly evolving field.

This narrative review will only include studies according to specific inclusion and exclusioncriteria. Considered studies will consist of primary research articles, reviews (utilized for searching primary studies), or book chapters available in every peer-reviewed academic range that speciallylook into the usage of pre-trained Man-made Intelligence models (BERT, ResNet, VGG and their variations) for prediction of ASCVD risk and categorization of ASCVD-associated states like plaque attributes or thickness. Studies published in a language other than English and without immediately accessiblefull texts will be excluded. In addition, relevant studies must describe their methods, results, and discussion of pre-trained AI models in ASCVD in adequatedetail. Studies that were either not based on pre-train AI models, do not specifically target atherosclerosis or its resulting events as pertained to CVD, or were abstracts, conference proceedings when full text was not provided, editorials, or nonacademicpublications will be excluded. Studies with major methodological limitationsor poor reporting of results and studies primarily describing baseline AI/ML methodologies without a clear bearing on ASCVD will also be excluded. Studies will first be screened by titles and abstracts for relevance,after which potentially eligible studies will undergo full-text screening to verify that all of the inclusion criteria have been satisfied. Such stringent conditions will assist ensure that this review summarizes the most related and high-quality educational literature that investigates the useof pre-trained AI models for ASCVD prediction and classification.

## Using Pre-Trained Artificial intelligence Modelsto Predict Atherosclerotic Cardiovascular Disease

The use of pre-trained AI, particularly in NLP, is a promising technology for predicting ASCVD risk or future cardiovascular events in patients with or at risk for ASCVD.^42^

A notable example is Med-BERT, which modifies the BERT architecture to pre-train context-aware models on structured EHR data.^5^ Trained on over 28 million EHRs, Med-BERT significantly outperforms models trained on larger datasets without pre-training, requiring only minimal fine-tuning (300-500 samples).^5^ The Med-BERT overcomes the challenge of limited data, a common barrier in medical research, by learning generalizable representations and efficiently transferring these findings to new prediction tasks.^43^

In another example, clinical BERT was used to classify statin nonuse and its reasons from unstructured EHRs across a diverse healthcare system.^44^ While focusing on statin adherence, clinical BERT’s ability to extract key medication adherence information from clinical text can inform models predicting cardiovascular outcomes. By identifying reasons for non-adherence, clinical BERT can contribute to more tailored interventions for ASCVD risk.^44^ Additionally, BERT was applied to predict secondary cardiovascular diseases in patients with metabolic diseases based on healthcare utilization patterns in health insurance claims data.^45^ This model achieved high AUC scores (around 97.9%), demonstrating BERT’s ability to capture complex relationships in healthcare data for accurate risk predictions.^45^ The BERT’s transformer-based architecture is well-suited for sequential data, learning disease progression and medication responses from sequences of diagnoses.

In a study comparing BERT and XLNet, XLNet outperformed BERT in predicting 6-month mortality among cardiac patients, suggesting XLNet’s superior ability to identify positive cases and predict irreversible outcomes like death.^45^ This comparison highlights that while BERT is effective, other transformer models like XLNet may have specific advantages for certain cardiovascular prediction tasks.

Many of these studies use EHRs as input, which include unstructured clinical notes, structured diagnoses, medication prescriptions, lab results, and healthcare utilization patterns. The BERT model is pre-trained on large text corpora or specialized clinical databases (e.g., MIMIC-III for Clinical BERT) and then fine-tuned for specific ASCVD prediction tasks. Common evaluation metrics include AUC-ROC, accuracy, precision, recall, F1-score, and additional metrics like the net reclassification index and the Brier score.

The BERT-based models for ASCVD prediction show high AUC scores exceeding 0.90, outperforming structured data-only approaches, particularly by leveraging unstructured clinical notes. However, challenges include the high computational resources needed, potential biases in EHR data, and difficulty interpreting predictions from these DL models.^46^ Additionally, model performance depends on the quality and diversity of training data.

Table 1 presents examples that highlight the diversity of AI models used in ASCVD prediction, demonstrating their potential to recognize previously unclassified patterns and enhance prognostic processes.

## Training Data on Atherosclerotic Cardiovascular Disease Classification UsingPre-Trained Artificial Intelligence Models

In recent years, studies have focused on utilizing pre-trained artificial neural networks, particularly convolutional architectures like ResNet and VGG, to interpret and classify various facets of ASCVD. These efforts include tasks such as characterizing atherosclerotic plaques, assessing arterial stenosis, and detecting rupture-prone plaques across different imaging modalities.

In image-based ASCVD classification, transfer learning with pre-trained ResNet-50 has yielded excellent results for detecting atherosclerotic plaques from CCTA.^14^ Studies show that fine-tuning ResNet-50 on medical image datasets achieves high recall, accuracy, and area under the receiver operating characteristic curve (AUC-ROC) scores. In many detection tasks, ResNet-50 has outperformed other CNN architectures like Inception-v3, VGG19, and baseline CNN models.^14^ Its residual connections are particularly effective in overcoming the vanishing gradient problem during deep network training, enabling the capture of fine visual patterns critical for precise medical image classifications.

Hybrid neural networks combining ResNet-50 and VGG-16 have achieved remarkable results, reaching accuracies near 99.45% for CAD detection and diagnosis.^3^ These ensemble models, which merge feature extraction strengths through techniques like Softmax voting, outperform individual models such as ResNet-50 or VGG-16 alone.^47^

For classifying carotid atherosclerotic plaques as vulnerable or stable based on ultrasound (US) images, models using a ResNet-50 backbone within a faster region-based CNN (Faster RCNN) have demonstrated high accuracy and reliability.^48^^,^^49^ The diagnostic performance of these models has been reported to rival that of expert physicians.^48^ Given that US is inexpensive and widely accessible, using ResNet-based models provides valuable support for early identification of high-risk plaques and stroke prevention.^50^ Beyond imaging, pre-trained CNNs like VGG16 have shown flexibility in other domains. For instance, VGG16 has been adapted to detect CAD from phonocardiogram (PCG) signals by learning patterns in acoustic data.^3^ This highlights the ability of CNNs to transfer learned features, such as edges and textures, to different modalities like frequency and amplitude data.

Additionally, fine-tuning VGG16 on mammograms has proven effective for detecting breast arterial calcifications (BAC), a female-specific biomarker for cardiovascular disease morbidity risk.^51^^,^^52^ The VGG16 not only outperformed deeper, more complex networks in F1-score but also provided more accurate localization of calcifications.^51^ Given the frequent use of mammography, integrating AI models like VGG16 could automate BAC detection and enhance early cardiovascular risk identification among women.^53^

The VGG19 has been among the pre-trained models benchmarked in comparative studies for diagnosing atherosclerosis using transfer learning on CT scans.^14^ While ResNet-50 outperformed VGG19 in that particular study, the inclusion of VGG19 highlights its frequent use in medical imaging research. Given the variability across imaging modalities and the importance of different performance metrics, it remains valuable to evaluate multiple pre-trained CNN architectures to identify the most suitable model for specific ASCVD classification tasks. Among the most commonly used imaging techniques for ASCVD classification is CCTA, where both ResNet-50 and VGG19 have been extensively fine-tuned. Adaptations of these models have also been applied to other datasets, such as intravascular US and OCT. These high-resolution, invasive imaging modalities support detailed plaque characterization, although explicit implementations of ResNet or VGG on these modalities are less commonly reported. Nevertheless, Faster RCNN models incorporating a ResNet-50 backbone have demonstrated superior AUC performance on such data. Ultrasound imaging has also been used in carotid populations to assess plaque presence and stability.

In parallel, BAC, a significant cardiovascular risk marker for women, have been effectively detected via mammography using transfer learning with VGG16. In another domain, CNNs pre-trained on ImageNet—like VGG16—have shown success in analyzing PCG signals to detect CAD, underscoring the flexibility of CNNs across both imaging and non-imaging modalities.

Popular pre-trained CNNs used for fine-tuning in these tasks include various ResNet architectures (e.g., ResNet-50) and VGG models (e.g., VGG16 and VGG19). Other architectures such as Inception and DenseNet have also been explored for similar purposes.^54^ These models support both binary classification tasks (e.g., distinguishing diseased vs. healthy arteries or stable vs. vulnerable plaques) and more complex multi-class classifications (e.g., identifying specific plaque components).

Overall, pre-trained CNNs have delivered promising results in ASCVD classification, with most studies reporting accuracies exceeding 90%. Performance can be further improved through ensemble methods that combine the strengths of different architectures. However, several challenges remain: the limited availability of large, high-quality annotated medical datasets; the variability in data across patients and imaging protocols; the interpretability problem inherent in “black box” DL models; and the risk of overfitting when using complex models on small datasets. In addition, the high computational demands of training and deploying DL models may limit their use in some clinical settings.

## Comparative Synthesis of Ascertainment Models for Pre-Trained ArtificialIntelligence in Atherosclerotic Cardiovascular Disease

The strengths and weaknesses of various pre-trained AI models, such as BERT, ResNet, VGG, and others, are often compared to assess their suitability for predicting and classifying ASCVD.

The BERT, designed for NLP, excels at analyzing sequential data, making it ideal for interpreting longitudinal EHRs and clinical notes. Its transformer architecture identifies contextual associations in clinical text, which can uncover hidden risk factors and improve understanding of treatment adherence. Because of its pre-training on large corpora, BERT can perform well even with small, domain-specific datasets. However, BERT is not suited for image analysis, such as plaque detection in imaging modalities, and its interpretability is limited due to the complexity of its architecture and the opaque nature of its attention mechanisms, especially when applied to varied and inconsistent EHR data.^55^ ResNet is a deep CNN optimized for image analysis. Its use of residual connections solves the vanishing gradient problem, enabling the training of very deep networks and making it highly effective for complex ASCVD-related tasks like plaque detection, stenosis quantification, and vulnerable plaque identification. Its hierarchical feature extraction yields high accuracy but comes with increased computational demands and lower interpretability. Large annotated datasets are typically needed, though transfer learning can ease this requirement.

The VGG, another popular CNN, has a simpler and more uniform architecture, making it easier to implement and modify. It has been successfully used for detecting CAD from PCG signals and BAC from mammograms. The VGG performs well on small to medium datasets but may struggle with complex features due to its shallower structure. Longer versions may still suffer from vanishing gradient issues, unlike ResNet.

Alternative architectures offer different advantages. Inception models are efficient and lightweight, DenseNet promotes feature reuse, making it suitable for smaller datasets, and EfficientNet balances depth, width, and resolution for high accuracy with optimized resources. Model choice depends on task complexity, data type, dataset size, and computational capacity.

For ASCVD risk prediction, BERT is ideal for leveraging structured EHR data and clinical notes. The ResNet is better suited for image-based assessments like CCTA or OCT, while VGG offers a balance of simplicity and performance, especially when resources or data are limited.

Successful clinical use of these models depends on high-quality, diverse data, model interpretability, and available resources. External validation, regulatory compliance, and seamless workflow integration are critical for bringing AI into routine ASCVD care.

## Discussion

Pre-trained AI models, in particular transformer-based models (e.g., BERT) for text and CNNs (e.g., ResNet, VGG) for images, demonstrate promising performance in predicting and classifying ASCVD. Yet, medical data high-dimensionality, sparsity, and heterogeneity pose challenges on clinical deployment. Transfer learning is increasingly being applied to fine-tune these models for medical purposes, despite not having well-labelled data for the tasks.

Large dataset pre-trained DL models have achieved great success in ASCVD-related tasks, such as EHR-based risk prediction, plaque detection and risk biomarker detection. So far, these models have been proven to be more accurate and diagnostic than conventional methods. Several developments are ongoing to improve pre-trained models, and fine-tuning methods and data integration from various sources are in progress.

Computer readers with pre-trained AI models may identify high-risk individuals before conventional predictors, enabling timely interventions and individualized treatments. Analysis of medical images using AI can be applied for better decision-making, to lessen cardiovascular outcomes, and as a means to automate daily, monotonous tasks, such as risk scoring that aids in enhancing the clinical value chain. Furthermore, AI-powered tools could deliver more unbiased and consistent evaluations that lead to better quality care. There are still some barriers to the use of AI in ASCVD management. If the training data is biased—meaning it overrepresents or underrepresents some demographic group or groups in relation to reality—this can result in biased predictions. It is important to have a diverse and representative dataset and to use fairness-aware techniques. Interpretability is also an issue—clinicians need to believe in the output of the AI in order to make clinical decisions. Methods for explainable AI (XAI) are crucial to increasing the transparency and user-friendliness of AI.

Proving and validating AI models are important, as they would be for any intervention. Most of the studies have been single-center and lack external validation, eventually leading to a possible lack of generalizability. Further work should focus on large, multicenter studies to maintain generalizability across subgroups of patients. Ethical and regulatory issues, including the patient privacy and algorithm accountability, also need to be properly solved. If AI is going to be integrated into the clinical workflow, it’s going to have to provide user-friendly interfaces, instant access to data and insights that clinicians can act upon at the point of care.

Future ASCVD investigation should emphasize interpretable models, utilizing XAI approaches to enhance explanation and merging datasets to mitigate biased results. Cross-modal learning methods that use text, imaging, or genomic data offer the potential for a hybrid approach to understanding ASCVD. Translational research to deploy AI tools into practice setting as well as studies to better understand their cost-effectiveness and clinical implications are required to maximize the use of AI for enhancing ASCVD outcomes.

## Conclusion

Across ASCVD, a wide range of pre-trained AI models—such as BERT for textual data and CNNs like ResNet and VGG for imaging—show promise in improving risk stratification, disease classification, and clinical workflow efficiency. However, their broad clinical adoption depends on overcoming key limitations: the need for large, high-quality, unbiased datasets; ensuring interpretability for clinical trust; and meeting external validation and regulatory standards. Future research should focus on addressing these challenges and evaluating the clinical utility and cost-effectiveness of such models. Successful integration into healthcare will require close collaboration between researchers, clinicians, and policymakers.

## Figures and Tables

**Table 1.** Comparison of AI Models for ASCVD Prediction

Model/Method	Modality	Performance Metrics
AI-ECG model	ECG	C-index 0.721, 5-year AUC 0.761, HR 2.33 (2.26-2.41) (Zhang et al, 2024)
CoroNet (Ensemble Deep CNNs)	Clinical data	Accuracy 83.61%, improved resilience with transfer learning (Juliet et al, 2024)
Transfer learning (VGG16, MobileNetV2, InceptionV3)	MRI	Test accuracies 97.94%-98.08% (Cyriac et al., 2022)
Deep CNN with Haar Wavelet decomposition	CCTA	Accuracy 95.2%, sensitivity 80% with CNN-KNN classifier (Laidi et al, 2022)
AI-CAC	CAC scans	AUC 0.826 for heart failure prediction, outperformed NT-proBNP and Agatston score (Naghavi et al, 2024)
PowerAI-CVD (CatBoost)	EHR data	AUC 0.869, significant prediction strength across subgroups (Li et al, 2023)

AI, artificial intelligence; AUC, area under the curve; CAC, coronary artery calcium; CCTA, coronary computed tomography angiography; CNN, convolutional neural network; CVD, cardiovascular disease; ECG, electrocardiogram; EHR, electronic health record.

## Data Availability

The data that support the findings of this study are available on request from the corresponding author.
